# *In vitro* reconstitution of biological oscillators

**DOI:** 10.3389/fcell.2025.1632969

**Published:** 2025-08-12

**Authors:** Ewan van der Vlist, Susan de Vries, Julia Kamenz

**Affiliations:** Molecular Systems Biology, Groningen Biomolecular Sciences and Biotechnology Institute, University of Groningen, Groningen, Netherlands

**Keywords:** biological oscillators, *in vitro* reconstitution, circadian clock, Min system, synthetic biology

## Abstract

Oscillations are fundamental to biological timekeeping and organization, yet understanding how their complex temporal dynamics emerge from underlying molecular interactions remains a significant challenge. *In vitro* reconstitution offers a powerful bottom-up approach to dissect the minimal components, interactions, and parameters required to generate these rhythmic behaviors. Biochemical reconstruction of minimal oscillators outside of their native cellular contexts allows the direct interrogation of the biochemical, biophysical, and systems-level properties that govern oscillatory dynamics and unravel the governing fundamental design principles. In this review, we summarize the theoretical foundations of biological oscillators and outline the major experimental challenges associated with their *in vitro* reconstitution. We highlight recent advances in the reconstitution of diverse oscillator types, including the cyanobacterial circadian clock, the Min system from *Escherichia coli*, and synthetic genetic oscillators such as the repressilator. These case studies illustrate how reconstitution efforts have yielded key mechanistic insights and driven technological innovation. We conclude by exploring emerging tools and future directions that promise to overcome current limitations and broaden the applicability of oscillator reconstitution–both to additional biological systems and to a wider range of scientific questions.

## 1 Introduction

Life on Earth is fundamentally rhythmic, shaped by oscillations that span vast ranges of time and length scales–from millisecond neuronal spikes and the human heart beating approximately once per second, to circadian clocks and multi-year predator-prey cycles (Li and Yang, 2018; Rapp, 1987). These diverse rhythms are generated and maintained by biological oscillators–regulatory networks that drive “cycles of change” (Winfree, 2001) in the abundances and activities of their molecular components and outputs, periodically returning the system to its starting point. Biological oscillators are ubiquitous across all kingdoms of life and are central to how organisms maintain internal order, keep time, and adapt to and anticipate changes of their environment. Perturbations to these endogenous oscillators can have widespread physiological consequences, disrupting processes such as development, metabolism, the immune system, hormonal signaling, and sleep cycles (Goldbeter, 2017). For example, chronic disruption of the circadian rhythm has been associated with an increased cancer risk in both mice and human (Stevens, 2009; Van Dycke et al., 2015), which has been suggested to stem from a desynchronization of the circadian clock and the cell cycle oscillator (Feillet et al., 2015). Hence, studying the molecular mechanisms and emerging system level properties of biological oscillators will not only reveal fundamental principles of life, but might also allow for the development of new interventions for these “dynamical diseases” (Glass and Mackey, 1979).

Oscillations can arise from relatively few chemical or biochemical interactions (Novak and Tyson, 2008); and although biological oscillators are typically embedded within large, complex regulatory networks, it is often possible to isolate a core oscillatory circuit composed of a limited number of components. For instance, while the eukaryotic cell cycle involves hundreds of proteins, its core oscillator can be reduced to just a handful of essential protein interactions (Ferrell et al., 2011). This manageable level of complexity lends itself to mathematical modeling, which has provided a robust theoretical framework to guide experimental investigation. *In vitro* reconstitution–the isolation of the oscillator from its endogenous cellular context–represents a logical next step for studying its biochemical and dynamical properties in a controlled environment. The bottom-up reconstitution of naturally occurring oscillators provides insights into how evolution has addressed the design challenges of generating oscillatory behaviors, identifies the minimal set of components required for sustained oscillations, and elucidates the molecular interactions that satisfy essential criteria imposed by theory. Furthermore, defined perturbations of reconstituted systems enable systematic exploration of dependencies, kinetics, dynamics, and boundaries not accessible to *in vivo* studies, thereby uncovering the design principles and emergent properties, e.g., robustness and tunability, of biological oscillators. Vice versa, design principles of biological oscillators can be applied to engineer novel to nature synthetic oscillator circuits. Leveraging a broad array of genetic and molecular tools such forward engineering via design-build-test-learn cycles can be used to empirically test theoretical models, and to address challenges and applications in synthetic biology and biotechnology (Brooks and Alper, 2021).

Biological oscillators can be classified in various ways, such as by network topology or biological context. For the purposes of this review, we categorize biological oscillators into three groups: (1) post-translational oscillators (PTO), which rely solely on protein–protein interactions and post-translational modifications; (2) membrane-bound oscillators, which additionally require spatial interactions with a membrane; and (3) transcription-translation oscillators–often referred to as genetic oscillators–composed of genes and their products. Each category presents distinct experimental challenges and has its own trajectory of discovery and success.

Here, we discuss recent advancements in the reconstitution of biological oscillators. We begin by discussing the theoretical requirements for oscillatory behavior, highlight relevant mathematical models, and point out key challenges associated with *in vitro* reconstitution. We then discuss several notable examples, including the reconstitution of naturally occurring oscillators, such as the cyanobacterial circadian clock and the Min system involved in bacterial division plane positioning, as well as synthetic transcription-translation oscillators such as the two-switch negative feedback oscillator and the repressilator. We highlight how these case studies have deepened our understanding of oscillator dynamics while driving both mechanistic insights and technical innovation in their respective fields. Finally, we offer an outlook on future directions and emerging technologies that may accelerate progress and unlock new opportunities in biological oscillator research.

## 2 Main text

### 2.1 Theoretical requirements for biological oscillators

Despite the seeming complexity of biological oscillators and their resulting fascinating temporal dynamics and spatial patterns, biological oscillations have been surprisingly amenable to mathematical modeling. Nonlinear dynamics, control theory and Bayesian statistics–among others–provide useful theoretical frameworks and tools to describe and interrogate the dynamic behaviors of biological oscillators (Ferrell et al., 2011). Rigorous abstraction and formalization of the experimental knowledge about a biological system enables the exploration of its dynamics and boundaries beyond the limits of human intuition and experimental feasibility, and allows for quantitative comparison of alternative network configurations and molecular mechanisms. The capacity for biological systems with (relatively few) molecular interactions to self-organize into higher order systems with system-level characteristics, e.g., period, amplitude, and robustness, has fascinated theoreticians for at least a century and has led to the continuous development of mathematical models alongside experiments–with theoretical predictions in many cases preceding experimental discovery.

Theory teaches us that despite the diversity in topology, time, and length scales of biological oscillators, for biological oscillations to occur a number of requirements need to be met. These requirements are (1) negative feedback, (2) time-delay, (3) nonlinearity, and (4) balanced timescales. To our knowledge, the review by Novák and Tyson (Novak and Tyson, 2008) is the first to systematically prove these four requirements, although some of these requirements were already suggested in earlier work (Friesen and Block, 1984; Goldbeter, 1997). Considering these theoretical requirements as well as their molecular implementations immediately highlights several of the experimental challenges that need to be overcome in order to successfully reconstitute biological oscillators.

#### 2.1.1 Requirement #1 - negative feedback

**FIGURE 1:**
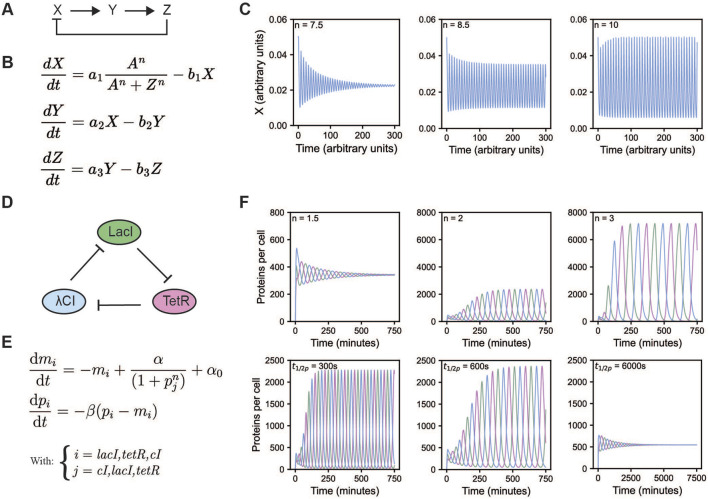
Overview of the Goodwin and repressilator model. **(A)** A schematic overview of the Goodwin model (Goodwin, 1965; Griffith, 1968). **(B)** Equations of the Goodwin model, with X, Y, and Z representing mRNA, an enzyme translated from that mRNA, and a metabolite produced from that enzyme, respectively. Here, *n* is the Hill coefficient. All *a* and *b* indicate scaling factors specific for that interaction. **(C)** Oscillations of the Goodwin model for different values of the Hill coefficient, initial values were set to 0.05, 0.2, and 2, for X, Y, and Z respectively, all other values were taken from Gonze and Ruoff (2021): *A* = 1, *a*_*1*_ = *a*_*2*_ = *a*_*3*_ = 5, *b*_*1*_ = *b*_*2*_ = *b*_*3*_ = 0.5. **(D)** A schematic overview of the repressilator. The repressilator is a negative feedback loop whereby each of the three proteins represses the transcription of the next protein in the cycle. **(E)** Equations of the repressilator model, with *m*_i_ being the mRNA concentration and *p*_i_ being the protein concentration of species i, *α*_0_ represents the number of transcripts per second made when the repressor is present in saturating amounts, while this is *α* + *α*_0_ in the absence of the repressor. *β* is the ratio of decay rates of protein to mRNA, and *n* the Hill coefficient. **(F)** Oscillations of the repressilator model for different values of the protein half-life (top row) and Hill coefficient (bottom row). To get the oscillations from the equations, rescaling is required, see (Åström and Murray, 2008; Elowitz and Leibler, 2000). The remaining parameter values were kept constant *α* = 0.5 and *α*_*0*_ = 5 * 10^–4^ transcripts/s, translation efficiency = 20 proteins/transcript, mRNA half-life = 120 s, K_m_ = 40 monomers per cell. Initial values where set to 1 and 20 for mRNA and protein levels of LacI, all others were set to 0. When protein half-life was varied, n was kept at 2. When n was varied, protein half-life was kept at 600 s.

Negative feedback is an absolute requirement for biological oscillations (Thomas, 1981). In fact, all biological oscillators described so far have a negative feedback mechanism at their core. This is in contrast to electronical systems that primarily rely on positive feedback for sustained oscillations (Tucker, 1972). Negative feedback is crucial to reset the system to its original state; if during a process the concentration of protein X increased, negative feedback is what will bring it down. Molecularly, a classic example of negative feedback is transcriptional repression. A protein might directly suppress the transcription of its coding gene, repress the synthesis of another protein present earlier in the reaction cascade, or inhibit the production of a protein within the same cyclic reaction pathway. One well known example is the circadian clock in *D. melanogaster*, where the period protein (PER) inhibits its own transcription (Hardin et al., 1990). The theoretical implementation is very similar to the Goodwin oscillator and explicit (see later discussion and Figure 1B). Here, PER (molecule Z) provides negative feedback on its mRNA production (molecule X). The importance of negative feedback in the circadian oscillator has also been proven theoretically, e.g., in the work on *Neurospora crassa* and *Drosophila melanogaster* (Smolen et al., 2001). Although negative feedback via gene repression is a common motif in cellular biological oscillators, negative feedback can also be implemented at the protein level–via product inhibition, protein degradation, or post-translational modifications–and even at the organismal level, such as predation in the classic Lotka-Volterra predator-prey system (Lotka, 1926; Volterra, 1926). During the eukaryotic cell cycle, negative feedback is present in a variety of ways, including the mitotic checkpoint sensing the physical attachment of microtubules to the kinetochores, and the core mitotic oscillator (Ferrell, 2013). Here, cyclin B activates the cyclin-dependent kinase 1 (CDK1), which in turn activates the anaphase-promoting complex/cyclosome (APC/C). The APC/C, an E3 ubiquitin ligase, then targets cyclin B for proteasomal destruction, allowing the system to return to its initial state of inactive CDK1 (Novak and Tyson, 1993). Many more examples demonstrating the importance of negative feedback for oscillations exist, e.g., work on the mitogen-activated protein kinase cascades (Kholodenko, 2000).

Whereas it seems intuitive that negative feedback is required for oscillations, it can also feel counterintuitive, since textbooks teach us that negative feedback is crucial for stability and homeostasis–driving the system back to its stable steady state if it diverges. How, then, can a negative feedback loop stabilize one biological system, yet cause continuous destabilization resulting in oscillations in another? The answer lies in the timing.

#### 2.1.2 Requirement #2 – sufficient time delay

Not every negative feedback will create oscillations, in fact, most will not. In order for negative feedback to be able to create oscillations, sufficient time delay is required. The time delay prevents the system from instantly returning to its initial state, causing it instead to more easily overshoot the target. When a state is continuously under- and overshot, oscillations arise. An everyday example for a time-delayed negative feedback oscillator is a difficult to adjust faucet. You increase the temperature, however, it takes some time for the water to heat up. Impatiently, you turn the temperature up more. Now, the water is scalding hot: You have overshot the desired temperature. Quickly you turn the temperature down, providing negative feedback. You may again go a bit too far, and end up at your starting point, with the water being too cold for your liking.

When modelling an oscillatory network, a trivial way of introducing time delay is by doing so explicitly. However, this does not aid in the understanding of how oscillations arise from the underlying molecular interactions. An implicit implementation of the time delay is therefore desirable. Molecularly, there are many possible sources for a time delay. For example, certain processes in the cell, such as transcription and translation or the physical movement of a protein or signal to a different location, are inherently time consuming and therefore prone to provide sufficient time delay. An example for such an inherently slow process is splicing. For Hes7 oscillations regulating somatic segmentation to occur, a time delay due to mRNA splicing was shown to be essential (Takashima et al., 2011). Multiple, intermediate steps in a signaling cascade or circuit can also result in sufficient time delay, which is how time delay is implemented in both the Goodwin oscillator and the repressilator–biological oscillator models which we will discuss later in more depth (Elowitz and Leibler, 2000; Goodwin, 1965).

Time delay and negative feedback together will get you a long way, but on their own they are not sufficient for sustained oscillations, as the system will eventually converge to its steady state (the same way you will eventually adjust the faucet to the right water temperature). To help the system to repeatedly move away from the steady state, some extra “force” is required.

#### 2.1.3 Requirement #3 – nonlinearity

Nonlinearity provides the push the system needs to get away from the steady state. In a nonlinear system a small perturbation (e.g., change in concentration) can result in big changes in the state of the system and thus destabilize the steady state. Again, this ties in nicely with our faucet example. It is easy to imagine that when a small rotation of the knob leads to a big temperature change, one can quickly overshoot the desired temperature in either direction. This effect is even worse if the knob shows additional nonlinear behavior–for instance, if it is a bit loose, and does not respond for the first few millimeters of movement. In that case, adjusting the temperature becomes nearly impossible as you desperately toggle from one temperature extreme to the other.

Nonlinearity can come from countless molecular processes; mechanisms that increase nonlinearity beyond Michaelis Menten kinetics include cooperative and allosteric binding (Goldbeter and Dupont, 1990), protein sequestration (Buchler and Cross, 2009), cascades (Huang and Ferrell, 1996), multisite phosphorylation (Gunawardena, 2005; Salazar and Hofer, 2009), autocatalysis (Goldbeter and Lefever, 1972) or even zero-order ultrasensitivity (Goldbeter and Koshland, 1981). In computational models, nonlinearity can be implemented via enzyme kinetics and cooperative binding modelled *via*, e.g., the Hill equation. The Hill equation is the source of nonlinearity in both the Goodwin oscillator and the repressilator, which we will discuss later (Figures 1B,E). Whereas in the Goodwin oscillator the molecular reasoning for the high Hill coefficient (n > 8) is unclear, for the repressilator the authors reasoned that the nonlinearity originates from the repression term due to the dimerization of the transcriptional repressors and cooperative binding to the repressor element (Elowitz and Leibler, 2000).

With negative feedback, time delay, and nonlinearity, almost all conditions for oscillatory behavior are met. However, achieving sustained oscillations critically depends on whether the timescales of the different biochemical processes align.

#### 2.1.4 Requirement #4 – balanced timescales

When one reaction runs much faster than the other, reaction products may deplete or accumulate, which disfavors oscillatory behavior (Novak and Tyson, 2008). Therefore, reaction partners need to have appropriately matching kinetic rates to reach the nonlinear part of their response. The repressilator model illustrates how changing the protein half-life–while keeping mRNA half-life constant–can promote or suppress oscillations (Figure 1F).

#### 2.1.5 Beyond the core requirements–Positive feedback

Finally, though not strictly required, positive feedback is a common feature of biological oscillators (Novak and Tyson, 2008). Positive feedback can provide strong nonlinearity and can therefore help with establishing sufficient time delay, alleviate the need for nonlinearity in individual molecular interactions, and contribute to balancing timescales, thereby supporting stable oscillations across a wide range of parameters (Ananthasubramaniam and Herzel, 2014; Tsai et al., 2008). Furthermore, oscillators with positive feedback loops are more tunable, meaning they retain a constant amplitude over a wide range of periods–a feature often desirable in biological systems. The tunability of your heartbeat, for example, is important to maintain a steady amplitude regardless of whether you are sleeping or running a marathon. Not surprisingly, a wide array of biological oscillators, spanning kingdoms, have been found to contain positive feedback loops. Robust circadian rhythms in *N. crassa,* for example, are mediated by interlocked negative and positive feedback between the white collar proteins WC-1 and WC-2 and FRQ (Cheng et al., 2001; Lee et al., 2000). Similarly, in the mammalian circadian clock there are additional positive interactions between RORalpha and Basic Helix-Loop-Helix ARNT Like 1 (BMAL1) (Akashi and Takumi, 2005; Nakahira et al., 2004). For a more complete overview of positive feedback loops present in biological oscillators, see the summary by Tsai et al. (Tsai et al., 2008).

All in all, theoretical models can greatly aid in our understanding of why and how biological systems oscillate and have allowed to define the requirements for oscillations to occur. Furthermore, they can help in exploring the parameter space, and thereby aid the reconstitution of naturally occurring oscillators as well as the design of synthetic biological systems including oscillatory ones. The repressilator ((Elowitz and Leibler, 2000), Figures 1D–F) is an exemplary case, where a simplified model enabled researchers to define key parameters required for oscillations, and use these theoretical insights to guide experimental implementation, as we will discuss in the next section.

### 2.2 Modeling biological oscillators

One of the earliest and most influential pieces of work regarding the modelling of biological oscillators is the work by Goodwin (Goodwin, 1963; Goodwin, 1965). His models were based on the discoveries of Jacob and Monod regarding gene regulation and transcription (Jacob and Monod, 1961). Originally, Goodwin devised a two-variable model, however, in this system the amplitude depends on the initial conditions. Further, if the system is perturbed, it changes amplitude instead of returning to its original values, which does not reflect the behavior of biological systems well. Therefore, the model was extended by an additional variable. The three-variable Goodwin model is a system described by three ordinary differential equations, as shown in Figure 1B (Goodwin, 1965; Griffith, 1968). There are several ways in which these equations can be interpreted, e.g., X, Y, and Z are commonly thought of as mRNA X, enzyme Y translated from X, and a metabolite Z produced by enzyme Y. Metabolite Z represses transcription of X forming negative feedback. Time delay is implicit in Goodwin’s model, and originates from the multiple steps in the regulation. Nonlinearity is introduced via a Hill function in the first equation, indicating cooperative repression of X. Only when the Hill coefficient, n, is larger than eight this particular system of equations shows oscillations (Figure 1C), which might not always be realistic for biological systems (Gonze and Abou-Jaoude, 2013; Griffith, 1968).

With the Goodwin model comprising the regulation of a single gene by its gene product, a logical next step is the addition of more genes to the system–yielding a circular network of repressors, also referred to as ring oscillators (Fraser and Tiwari, 1974). The first successful reconstruction of such a network in *Escherichia coli* was the so-called “repressilator” from Elowitz and Leibler ((Elowitz and Leibler, 2000), Figures 1D, 2). In their work, both a mathematical model of the repressilator as well as the *in vivo* implementation were presented. The implemented repressilator constitutes a three-gene network consisting of the Lactose operon repressor (LacI) from *E. coli*, the tetracycline repressor (TetR) from the Tn10 transposon, and the transcriptional repressor cI from the *λ* phage. The three genes are linked in a negative feedback loop where LacI represses the transcription of *tetR*, TetR represses transcription of *cI,* and cI represses the transcription of *lacI* (Figure 1D). Green fluorescent protein (GFP) was used as a reporter, its promoter repressible by TetR. The design of the repressilator started from a simplified mathematical model, made to identify conditions that would generally lead to oscillations. The model consists of six coupled first-order differential equations, with p_i_ and m_i_ representing protein and mRNA concentrations, respectively (Figure 1E). The other parameters include: The amount of mRNA produced from the promoter when bound by the repressor (α_0_), the amount of mRNA produced from the promoter when unbound (α), the ratio of decay rates of protein to mRNA (β), and the Hill coefficient (n). Sensitivity analysis of the repressilator model showed that oscillations were favored under strong promoters, tight transcriptional repression, repression being cooperative, and balanced decay rates of protein and mRNA. To increase chances of oscillations, these predictions were taken into account in their experimental design: Strong, tightly repressible promoters were used as well as protease-targeting ssrA-tags to shorten the lifetime of repressor proteins, bringing the lifetime closer to that of the corresponding mRNA. Similar to the Goodwin model, time delay arises from the multiple steps in the reaction network and is thus implicit. Nonlinearity comes from the Hill function in the repression term. An important distinction from the Goodwin oscillator is that the repressilator model can generate oscillations with a significantly lower Hill coefficient of two (Figure 1F), a value more physiologically reasonable and justified by the dimerization and cooperative binding of the transcriptional repressors to DNA.

**FIGURE 2:**
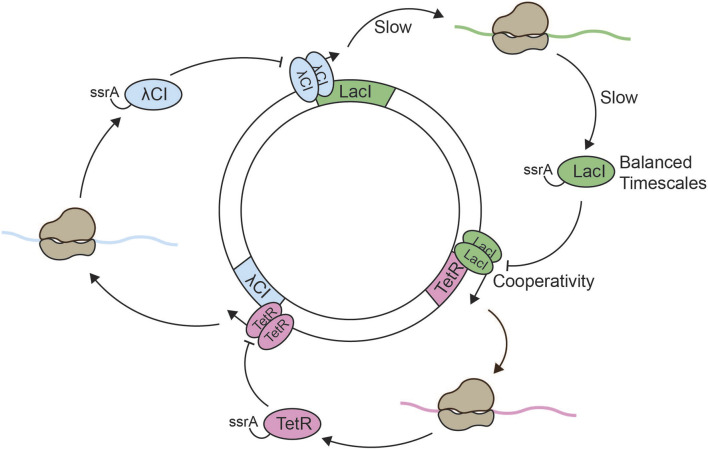
Reconstitution of the repressilator. Schematic overview of the *in vitro* repressilator system consisting of the *lacI, λcI,* and *tetR* genes on a single plasmid (Elowitz and Leibler, 2000; Niederholtmeyer et al., 2015). Expression of the *lacI* gene leads to the production of ssrA-tagged LacI. Dimerized LacI inhibits the expression of *tetR*. Expression of the *tetR* gene leads to the production of ssrA-tagged TetR, and dimerized TetR inhibits the expression of *λcI*. Expression of the *λcI* gene leads to production of ssrA-tagged λCI, and dimerized λCI inhibits the expression of *lacI*. Negative feedback arises from the odd number of inhibitory nodes, which results in *lacI* expression leading to *lacI* repression. The slow processes of transcription and translation establish sufficient time delay in the system. Nonlinearity is obtained through dimerization of the repressors and cooperative binding to the two repressor sites in each promoter. The addition of ssrA-tags is optional, but increases the degradation rate which generates oscillations with longer period and lower amplitude under otherwise constant conditions.

The original repressilator design *in vivo* showed sustained oscillations with a period of approximately 150 min. However, only about 40% of the bacteria showed oscillations. Biological noise as well as the interference between the endogenous *E. coli* environment and the repressilator components might have contributed to the stochasticity in the observed oscillations. Thus, the study highlighted both the great potential of synthetic circuit design as well as the challenges of designing robust oscillators *in vivo.* Later works have significantly improved the *in vivo* repressilator. For example, increased regularity and robustness were achieved by ensuring constant experimental conditions, e.g., continuously providing fresh medium, and equalizing the binding strengths of all repressors (Potvin-Trottier et al., 2016).

### 2.3 Challenges of reconstituting biological oscillators

Since the development of early theoretical models like the Goodwin oscillator and the implementation of the repressilator, a wide variety of oscillator designs have been explored *in silico* and successfully engineered in living systems, including bacteria, yeast, and mammalian cells (Purcell et al., 2010; Li and Yang, 2018). In contrast, bottom-up reconstitutions of biological oscillators have only been successful in a handful of cases. The power of *in vitro* reconstitutions lies in their ability to define the minimal set of components and precisely map the interactions, rates, and parameters required for oscillatory dynamics. However, identifying this minimal configuration and navigating the appropriate parameter space are inherently difficult tasks. When the reconstituted system fails to oscillate, it is often unclear whether this is due to a missing component, missing nonlinearities, imbalanced concentrations or stoichiometries, or suboptimal buffer conditions. The space of experimental variables can be insurmountably large, and systematically optimizing even a few of these variables can be tedious and time consuming. In the following sections, we will explore some key challenges in the reconstitution of biological oscillators–many of which are tightly connected to the theoretical requirements discussed earlier–and highlight recent efforts to address them.

#### 2.3.1 Hidden components

Unlike naturally occurring oscillators and synthetic oscillators established *in vivo* which utilize the cell’s basic molecular machinery, *in vitro* reconstitution must account for the absence of such endogenous factors. Consequently, the list of components may be significantly longer, and the interactions and dependencies far more complex to reconstitute than initially suggested by a simple network diagram or mathematical model, where synthesis and degradation terms are easily added and modified with the stroke of a pen. For certain oscillators, such as genetic oscillators, these “hidden” components can make up a substantial portion of the system and pose considerable hurdles.

##### 2.3.1.1 Transcription and translation

Biological oscillators requiring mRNA and protein synthesis necessitate the reconstitution of not only regulatory interactions, but also the complete transcription and translation machinery. Cell-free protein synthesis (CFPS) systems have become the preferred tool in these cases. Over the past two decades, protein yields from CFPS systems have significantly improved–both for CFPS based on crude lysates (Caschera and Noireaux, 2014; Jia et al., 2017), as well as for the fully reconstituted PURE (Protein synthesis Using Recombinant Elements) system (Kazuta et al., 2014; Shimizu et al., 2001). However, several challenges remain, including complex resource dependencies and limitations, e.g., adenosine triphosphate (ATP) and nucleoside triphosphates (NTPs) as mRNA precursors, and amino acids (Nagaraj et al., 2017) as protein precursors, as well as the accumulation of toxic reaction byproducts, e.g., inorganic phosphate (Kim and Swartz, 2000), which limit transcription-translation activity to just a few hours in the absence of continuous dialysis (Kazuta et al., 2014). Furthermore, control and finetuning of protein concentrations and stoichiometries continue to be difficult in these systems and require optimization on a case-by-case basis (Murakami et al., 2019; Ramm et al., 2022).

CFPS reactions based on crude cell lysates usually are more cost effective and provide higher protein yields compared to completely reconstituted systems such as PURE. Furthermore, with their protein-rich crowded environment and presence of numerous protein chaperones they can better promote protein folding, and, depending on the source, promote post-translational modifications. Crude lysates based CFPS systems from a variety of sources including *Saccharomyces cerevisiae* (Khattak et al., 2014; Rasor et al., 2021), mammalian cell lines such as HeLa cells or CHO cells (Kopniczky et al., 2020; Thoring et al., 2017), and chloroplast-based lysates from several plant species (Bohm et al., 2024) have been established. The diversity in CFPS systems can benefit reconstitution of biological oscillators by selecting the CFPS system that best matches oscillator specific requirements. *E. coli* based crude lysates have had the longest history of optimization for expression yields, whereas *S. cerevisiae* lysates have been primarily used to study metabolic pathways that are easily manipulated and well conserved (Rasor et al., 2021). Mammalian cell lysate systems have typically been developed to facilitate difficult to express or to fold proteins as they possess extensive refolding machinery, whereas chloroplast-based lysates have been primarily used for gene expression regulation by light or other inducible stimuli (Bohm et al., 2024; Kopniczky et al., 2020). For a more detailed, application-focused discussion of CFPS systems we refer to Gregorio et al. (Gregorio et al., 2019).

Despite their many advantages, crude lysates also have disadvantages, such as non-specific nuclease and protease activities, as well as unpredictable and difficult-to-control interactions between oscillator components and the host system. It is these kinds of interactions reconstitution efforts usually try to circumvent. The PURE system, utilizing a defined set of recombinant components, solves some of these issues, but suffers from comparably low yields and impaired translation processivity, leading to premature termination (Doerr et al., 2021), and resulting in significant reductions in its protein synthesis capacity. These current limitations of CFPS systems continue to pose considerable obstacles for reconstituting transcription-translation-based oscillators.

##### 2.3.1.2 Resetting the clock

Negative feedback is essential for biological oscillators to reset and return to their initial state. This reset requires reversing many of the reactions that previously moved the system away from its starting point. For a successful reconstitution, such resetting–like transcription and translation–necessitates additional “hidden” components and complex molecular machineries beyond direct inhibitory interaction or transcriptional repression. For instance, protein and mRNA synthesis must be counteracted by degradation, and post-translational modifications like phosphorylations must be reversed by enzymes such as phosphatases. Frequently, these counteracting enzymes are unknown and need to be identified first.

For cell-free protein synthesis, systems and reactions have been significantly optimized throughout the years, however, the reset reactions have received less attention. In fact, CFPS systems based on *E. coli* lysates are commonly prepared from strains with low protease activity (Caschera and Noireaux, 2014). Nevertheless, *E. coli*-derived extracts exhibit intrinsic mRNA and protein degradation activity, albeit at lower rates than *in vivo*, partially due to the diluted nature of the extract (Shin and Noireaux, 2010). Improvements have been achieved by supplying the extracts with additional regulators and enzymes. For example, CFPS lysates expressing, or supplemented with, MazF, a small sequence-specific ribonuclease, have been shown to increase the rate of mRNA inactivation and decrease the resulting protein yield in the lysate in a dose-dependent manner (Bartelds et al., 2023; Garamella et al., 2016; Shin and Noireaux, 2010). Similarly, the AAA + protease ClpXP, when supplemented into bacterial CFPS or expressed in the PURE system, enables targeted protein degradation for substrates fused to specific degradation tags such as ssrA (Garamella et al., 2016; Niederholtmeyer et al., 2013; Shi et al., 2018; Shin and Noireaux, 2010). MazF and ClpXP therefore currently constitute well-characterized options for implementing mRNA and protein degradation.

Nonetheless, significant hurdles continue to exist. Energy consumption for ClpXP-mediated protein degradation varies widely depending on the stability of the protein, but can require up to 500 ATP molecules for a model substrate, Titin-I27-ssrA, consisting of 121 amino acids (Kenniston et al., 2003). This issue is not limited to ClpXP: Whereas increased turnover–be it protein synthesis and degradation, phosphorylation-dephosphorylation cycles, or other ATP-dependent processes–is desirable to allow a system to be dynamic and responsive (Gelens and Saurin, 2018), it starkly increases the energy demand on the system. These higher demands need to be accommodated either by increased ATP concentrations, the presence of an ATP regenerating system, or both. However, high ATP concentrations are not always compatible with other activities in the lysate and need to be carefully adjusted and buffered, e.g., to not chelate Mg^2+^ from the system (Shi et al., 2018). Furthermore, while ClpXP enables protein degradation, its expression in the PURE system has been shown to suppress overall mRNA expression and reduce protein yield (Niederholtmeyer et al., 2013). The cause for this repression remains unclear, but underscores the complex and often unpredictable adverse interactions between individual components which complicate reconstitution efforts. Further improvement of activity, controllability, and compatibility of these approaches are essential to be able to employ them in bottom-up reconstitutions of biological oscillators.

#### 2.3.2 Nonlinearities

Nonlinearity is a fundamental requirement for oscillations to occur. This behavior can arise through various mechanisms, such as cooperative interactions at the molecular level, or positive feedback and zero-order ultrasensitivity at the network level. However, in many natural oscillators, the sources of nonlinearity are not immediately apparent. In some cases, sufficient nonlinearity may result from the combined effect of multiple weak nonlinear interactions. For example, it has been suggested that multisite phosphorylation of the APC/C by CDK1 could provide the necessary nonlinearity and time delay for the core embryonic cell cycle oscillator to sustain oscillations, although this has not yet been tested *in vitro* (Yang and Ferrell, 2013). Biochemical reconstitution offers a promising approach for mapping such interactions and uncovering the underlying sources of nonlinearity. Despite these insights, it remains challenging to determine whether a lack of nonlinearity is truly responsible for the absence of oscillations in a reconstituted system, or whether other factors are involved. Furthermore, even when insufficient nonlinearity is identified as the limiting factor, it is not always straightforward how to address this. For synthetic oscillators, researchers can draw on extensive genetic and molecular toolboxes to fine-tune nonlinearities or introduce additional network links (Garenne et al., 2021). By contrast, resolving such issues in natural oscillators may require the identification of missing components–a more complex and less tractable task.

#### 2.3.3 Balancing time scales and finding the correct parameter space

Oscillations typically emerge only within a narrowly defined parameter range, where the kinetic rates and concentrations of all components–enzymes, substrates, and metabolites–must be precisely balanced (Novak and Tyson, 2008). Identifying these critical experimental parameters within a large, high-dimensional space remains a major challenge. Mathematical models can contribute to our qualitative and quantitative understanding of such systems, help narrow down possibilities, and therefore play a crucial role in guiding experimental design. However, these models often rely on sparse experimental data, leading to significant uncertainties in the estimation of kinetic parameters (Pitt and Banga, 2019) – uncertainties that leave orders of magnitude to be experimentally evaluated. Careful biochemical characterization of individual enzymatic reactions or small reaction modules can help alleviate this issue by constraining parameter estimates and improving model accuracy. In addition, understanding how parameters change under changing experimental conditions (e.g., ionic strength, temperature, pH, crowding) is essential to identify buffer conditions compatible with all reactions constituting the oscillator. However, such analyses can be incredibly time consuming and labor intensive; and even with such characterization at hand, unpredictable interactions of the different system components are likely to remain. Therefore, the capacity of screening experimental conditions with high throughput methods and semi-automatic analysis tools are desirable, but often require cutting-edge instrumentation such as microfluidic systems, microliquid dispensers, fluorescence-activated sorting, and high-end microscopes, as well as experimental designs compatible with these methods. Hence, parameter estimation–both experimentally and *in silico*–remains a major challenge in the reconstitution of biological oscillators.

### 2.4 Examples of successfully reconstituted biological oscillators

Despite all these challenges, researchers have succeeded in reconstituting a number of important biological oscillators, and these achievements can be used as valuable lessons of what it takes to succeed as well as what can be gained from such efforts.

#### 2.4.1 Reconstitution of a post-translational oscillator: the cyanobacterial circadian clock

**FIGURE 3:**
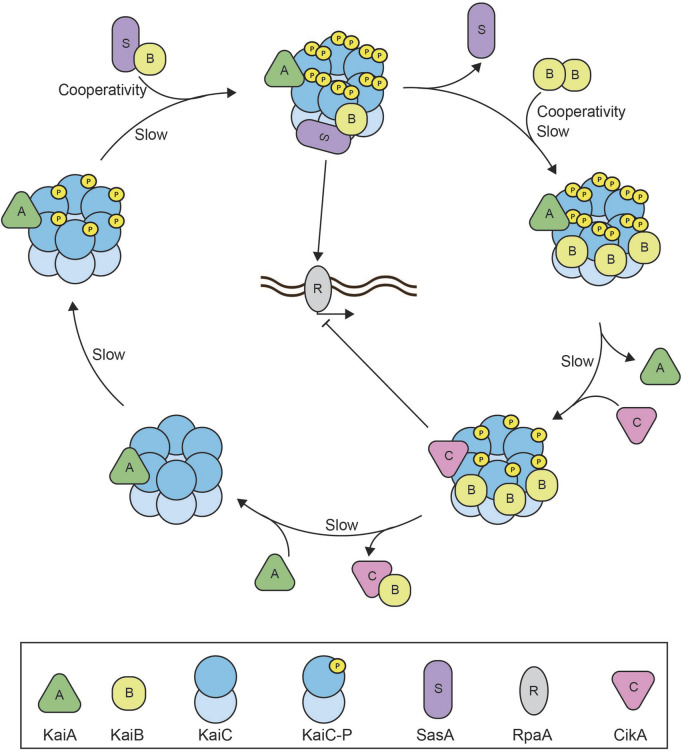
Reconstitution of the cyanobacterial circadian clock. Schematic overview of the reconstituted cyanobacterial circadian clock consisting of the core proteins: KaiA, KaiB, and KaiC, the histidine kinases SasA and CikA, and transcription factor RpaA (Chavan et al., 2021). The system oscillates with an approximately 24 h rhythm of KaiC phosphorylation. At dawn, KaiA will bind to the hexameric KaiC complex to facilitate KaiC autophosphorylation at two different residues. SasA binds to the KaiA-KaiC(P) complex to perform downstream activation of RpaA and global gene expression. SasA also aids KaiB binding to the KaiC complex and triggers cooperative binding of subsequent KaiB monomers until six monomers of KaiB have formed the KaiA-KaiB-KaiC(P) complex at dusk. The full stoichiometric balance between KaiB and KaiC causes the release of KaiA, binding of CikA, and facilitates KaiC autodephosphorylation. Downstream CikA inhibits RpaA to inhibit global gene expression. CikA and KaiB are released from the complex once KaiC is dephosphorylated and the cycle is completed. The negative feedback arises as KaiA binding to KaiC stimulates KaiC activation and autophosphorylation which eventually faciliates KaiA dissociation and KaiC dephosporylation. Slow kinetics of both the KaiC ATPase as well as KaiB association with KaiC introduce sufficient time delay (Egli et al., 2012; Kawamoto et al., 2020). SasA and KaiB binding to the complex generate cooperativity within the oscillator (Hong et al., 2020).

The earliest and ultimate success story of bottom-up reconstitution of a biological oscillator is the *in vitro* reconstitution of the circadian clock of the cyanobacterium *Synechococcus elongatus* (Nakajima et al., 2005) (Figure 3). The cyanobacterial circadian clock consists of three proteins: KaiA, KaiB, and KaiC, that are essential to establish rhythmic day-night cycles in gene expression of at least 30% of *Synechococcus* genes (Ishiura et al., 1998; Ito et al., 2009). Together, the Kai proteins generate an approximately 24-h rhythm of KaiC phosphorylation and dephosphorylation *in vivo* (Tomita et al., 2005). During the day phase of the oscillator, KaiA stimulates the ordered phosphorylation of KaiC at residues S431 and T432 (Nishiwaki et al., 2007; Nishiwaki et al., 2004; Rust et al., 2007; Xu et al., 2004). During the night phase, KaiB suppresses KaiC phosphorylation (Kitayama et al., 2003; Xu et al., 2003) leading to KaiC dephosphorylation. The KaiC phosphorylation state is translated into downstream phosphorylation of RpaA by two opposing sensor histidine kinases, SasA and CikA. The binding of SasA to KaiC enables autophosphorylation of SasA. SasA subsequently transfers this phosphoryl-group to RpaA. In its phosphorylated state, RpaA regulates a set of circadian effectors that orchestrate genome-wide gene expression (Ito et al., 2009; Markson et al., 2013; Takai et al., 2006). CikA on the other hand removes the phosphoryl-group from RpaA and transfers it onto itself. The fate of this phosphoryl-group from CikA-P has remained unknown (Gutu and O'Shea, 2013).

The cyanobacterial circadian oscillator was first reconstituted in 2005 (Nakajima et al., 2005) using recombinant KaiA, KaiB, and KaiC purified from *E. coli* in the presence of ATP, which is required for KaiC autophosphorylation. The system oscillated with an approximately 24-h period for at least 72 h. This unequivocally demonstrated that the interactions between these three proteins are sufficient to establish autonomous oscillations in KaiC phosphorylation closely resembling those *in vivo*. Moreover, the minimal oscillator exhibited important biological characteristics of the cyanobacterial clock such as temperature compensation–the ability to insulate period and amplitude from environmental temperature fluctuations. A temperature increase from 25°C to 35°C only minimally reduced the period from 22 h to 20 h, yielding a Q_10_ coefficient of 1.1 for thermal sensitivity of the period, similar to the Q_10_ coefficient determined *in vivo* (Nakajima et al., 2005; Kondo et al., 1993). That said, several observations also suggested limitations to the reconstitution: The KaiC phosphorylation rhythm had a lower amplitude than observed *in vivo* and showed a slight dampening, suggesting imbalances in the biochemical reactants. In addition, the frequency of the *in vitro* oscillations was higher than that *in vivo* (Nakajima et al., 2005). These differences between the *in vitro* reconstitution and the *in vivo* circadian clock may have been due to suboptimal stoichiometry of Kai protein concentrations or the absence of additional regulatory components.

The optimal stoichiometry was later identified through titration experiments of KaiA and KaiB, which demonstrated that the period and amplitude of oscillations in KaiC phosphorylation are sensitive to KaiA concentrations, but not KaiB, although both proteins need to exceed a defined threshold in order to sustain oscillations (Nakajima et al., 2010; Kageyama et al., 2006). For example, the KaiC hexamer requires a full stoichiometric complement of KaiB to stably sequester KaiA and form the night complex (Nakajima et al., 2010). These findings highlighted the importance of precise Kai protein concentrations for the oscillation rhythm. In addition, the roles of the two regulatory histidine kinases, SasA (Gutu and O'Shea, 2013) and CikA (Kaur et al., 2019), were characterized and shown to impact the KaiC phosphorylation rhythm by stabilizing KaiA:KaiC complexes and by stimulating the transition to KaiB:KaiC complexes (Kawamoto et al., 2020).

Based on these findings and a more detailed understanding of the KaiC phosphorylation mechanism (Kitayama et al., 2013), an improved and extended *in vitro* reconstitution of the circadian clock was achieved in 2021 (Chavan et al., 2021). To allow for the investigation of the downstream dynamics of the KaiC phosphorylation cycle, the extended reconstitution included the two histidine kinases, SasA and CikA, as well as the transcription factor RpaA and a DNA duplex as the RpaA target. The reconstitution was further facilitated through technological advances in monitoring the clock dynamics. Originally, the oscillations were analyzed by separating KaiC phospho-states using sodium dodecyl sulfate–polyacrylamide gel electrophoresis (SDS-PAGE) (Nakajima et al., 2005), which is time consuming and labor intensive. To increase the throughput of the circadian clock measurements, the authors conjugated fluorescent probes to each component of the circadian clock (Heisler et al., 2019). Measuring fluorescence anisotropy, which increases when the fluorescently labeled component binds to the large KaiC complex, allowed them to monitor the rhythmic dynamics of KaiC complex formations with the different regulators in real time and with increased throughput. By identifying the optimal stoichiometric balance for oscillations, the authors were able to observe sustained, undampened oscillation over the entire course of the experiment (up to 192 h) (Chavan et al., 2021).

Previous titration experiments had demonstrated the oscillator’s sensitivity to KaiA and KaiB concentration, but the novel methodology enabled a more extensive characterization of the impact of Kai protein stoichiometry for the oscillations. The new measurements confirmed the originally identified sensitivities and further defined a narrow concentration range permissible for oscillations (Chavan et al., 2021; Nakajima et al., 2010). KaiA titration showed that the system only oscillates below a 1.5-fold excess of KaiA, whilst a shortage of KaiA gradually decreases amplitude and increases the period of the oscillations (Chavan et al., 2021). This is consistent with *in vivo* studies that showed that KaiB and KaiC abundances are at least two-fold higher than KaiA abundance (Chew et al., 2018). KaiB is required at equal concentration to KaiC to observe oscillations, but increasing KaiB concentration beyond this does not affect the amplitude or period (Chavan et al., 2021). This aligns with the finding that KaiB is in slight excess over KaiC *in vivo* (Kitayama et al., 2003).

The reconstitution of the cyanobacterial circadian clock revealed multiple mechanistic details that allow the system to function as a biological oscillator. First of all, the circadian period as well as the KaiC phosphorylation cycle are driven by KaiC ATPase activity, an unusually slow process (Egli et al., 2012). The phosphate group released from ATP hydrolysis by KaiC is transferred to KaiC itself as a means of autophosphorylation, which is suggested to reduce the ATPase activity (Terauchi et al., 2007). The intrinsic KaiC ATPase activity (∼15 ATP per day) is very low compared to similar ATPases (10^3^–10^7^ ATP per day), which contributes to the ∼24 h period of the auto (de)phosphorylation cycle (Terauchi et al., 2007). The KaiC ATPase activity is positively and negatively regulated by the KaiA:KaiC complex and KaiB:KaiC complex, respectively, which are temporally segregated. The day-to-night transition requires the recruitment of six KaiB monomers, whereas gradual release of KaiB drives the night-to-day transition (Chavan et al., 2021; Nakajima et al., 2010). The kinetics of these complex formations are relatively slow with an approximate rate of 0.3–0.5 h^−1^ (Kawamoto et al., 2020; Mukaiyama et al., 2018). Together, the slow KaiC ATPase activity and the relatively slow kinetics of KaiA:KaiC and KaiB:KaiC complex formations provide two separate characteristics that establish sufficient time delay for the oscillator.

Secondly, KaiA and KaiB both demonstrate different degrees of nonlinearity within the Kai system. KaiA has an ultrasensitive dependence on KaiC phosphorylation, which is derived from differential affinities of KaiA for different nucleotide-bound states of KaiC (Hong et al., 2020). Also, KaiA sequestration by KaiB, which requires the recruitment of six monomers of KaiB to drive the day-to-night transition, occurs in a nonlinear fashion through cooperative binding (Hong et al., 2020). Altogether, the interplay between the different proteins temporally separates the activities from KaiC in a nonlinear manner to generate the autonomous oscillation.

Finally, the period of the circadian clock is affected by ATP/Adenosine diphosphate (ADP) ratios. Higher ATP/ADP ratios shorten the period and abrupt changes in the ATP/ADP ratio cause substantial phase shifts probably due to ADP competitively inhibiting KaiC’s autophosphorylation reactions. ATP/ADP concentrations raise during the day due to the active photosynthesis. Therefore, the sensitivity to the ATP/ADP ratio could be one mechanism by which the cyanobacterial circadian clock is entrained by its environment (Rust et al., 2011).

The example of the *S. elongatus* cyanobacterial circadian clock shows the success of *in vitro* reconstitution of a cytoplasmic oscillator, however also highlights its challenges. The Kai system requires energy in the form of ATP and oscillations are sensitive to ATP/ADP ratio, protein concentrations, and complex stoichiometry. Early reconstitution attempts generated autonomous, yet dampened, oscillations (Nakajima et al., 2005). The challenge to identify additional regulatory components and appropriate protein concentrations had to be overcome for the successful reconstitution. Crucially, the latest system closely resembles the *in vivo* behavior of the oscillator (Chavan et al., 2021) and shows sustained, undampened oscillations for more than a week. However, many questions remain for the Kai system. For example, how Kai protein homeostasis, including additional expression and protein degradation, impact the oscillation rhythm remains unclear (Cohen et al., 2018; Kitayama et al., 2003). In this context, implementing the feedback of the circadian clock onto the expression of Kai proteins would be interesting, but will require the reconstitution of the Kai oscillator in a transcription-translation system (Takai et al., 2006). In addition, recently discovered proteins, LabA and KidA, have been shown to regulate cyanobacterial circadian clock components and should be incorporated into the reconstitution to characterize their effects on the oscillation rhythm (Kim et al., 2022; Taniguchi et al., 2007).

*In vitro* reconstitutions have also given insights into the evolution of circadian rhythms. Whereas the cyanobacterial clock derived from the *S. elongatus* PCC7942 (*Se*7942) strain has been the earliest and best studied example of a Kai-protein based oscillator, recent studies have reconstituted a variety of Kai-protein oscillators from other organisms. A large-scale genome analysis identified double-domain KaiC homologs in different species, including other cyanobacteria, other bacteria, and even archaea (Dvornyk et al., 2003; Mukaiyama et al., 2025). Based on this analysis, oscillations could be successfully reconstituted using KaiA, KaiB, and KaiC from numerous freshwater and marine cyanobacteria. In contrast, no Kai proteins derived from other bacteria or archaea where able to sustain oscillations. Furthermore, ancestral KaiC variants were reconstructed based on branching points in the phylogenetic tree, allowing the reconstitution of several ancestral oscillators–but only for cyanobacterial nodes, not for more ancient, pre-cyanobacterial lineages (Mukaiyama et al., 2025). Interestingly, one of these ancestral biological oscillators–hypothesized to have appeared around 2.2 billion years ago–could be temperature-entrained to an 18 h period at pH 7.0. This is consistent with an ancestral cyanobacterial clock accommodating a significantly shorter day-night cycle. For example, 0.95 billion years (Gyr) ago, when cyanobacteria had already been present for approximately 2.5 Gyr, the Earth’s day was ∼18 h long due to its faster rotation, driven by its gravitational interaction with the moon and the planet’s mass distribution (de Sitter, 1927; Schopf and Packer, 1987; Scrutton and Hipkin, 1973). Similarly, a recent study also reconstructed and expressed ancestral counterparts (anKaiABC) of KaiABC based on phylogenetic analysis (Li et al., 2025b). Introducing *ankaiABC* into a *kaiABC* null strain of *Synechococcus* led to detectable rhythms under both 9-h light/9-h dark (LD9:9) and LD12:12 conditions. Furthermore, after 13 days of LD9:9, the growth rate of the *ankaiABC* strain was significantly higher than that of the *kaiABC* strain. This indicates that the ancient clock can be entrained by light-dark cycles and was adapted to 18 h days. These differences in periodicity arise from structural differences between anKaiABC and the extant KaiABC. Specifically, ancestral KaiC proteins seem to have a lower ATPase activity, kinase activity, and phosphatase activity (Li et al., 2025b; Mukaiyama et al., 2025). Furthermore, the capability of ancestral KaiA and KaiB to control KaiC’s phosphorylation status is impaired. Altogether, these studies demonstrate the power of *in vitro* reconstitution as a tool to dissect the evolutionary history and mechanistic diversity of biological oscillators across large phylogenetic distances.

#### 2.4.2 Reconstitution of a membrane-bound oscillator: the MinD-MinE system

Another post-translational biological oscillator, which has been successfully reconstituted *in vitro*, is the pole-to-pole oscillator found in *E*. *coli*. In contrast to the cyanobacterial circadian clock, which functions as a well-mixed system, spatiotemporal regulation of the pole-to-pole oscillator components shuttling between the cytoplasm and the membrane is crucial for the functionality of this standing wave oscillator (Hu et al., 2002). The pole-to-pole oscillator consists of two proteins, MinD and MinE. ATP-dependent interactions between MinD, MinE, and the plasma membrane establish a MinD gradient that is highest at the poles and lowest in the middle of the rod-shaped cell (Hu and Lutkenhaus, 1999; Raskin and de Boer, 1999b; Hale et al., 2001; Meinhardt and de Boer, 2001; Raskin and de Boer, 1999a). In addition to its interaction with MinE, MinD also recruits MinC. Although MinC is not an intrinsic component of the oscillator, it does act as a downstream effector by inhibiting Z-ring assembly at the poles, where MinC concentration is highest (Dajkovic et al., 2008). In that way, the oscillations of MinD restrict Z-ring assembly to the center of the cell, ensuring symmetric cell division.

*In vitro* reconstitution of the Min system resulted in various patterns (Ivanov and Mizuuchi, 2010; Loose et al., 2008; Vecchiarelli et al., 2014; Zieske and Schwille, 2014). Patterns ranged from amoebas, circular MinD zones surrounded by MinE, to travelling waves or bursts (Caspi and Dekker, 2016; Mizuuchi and Vecchiarelli, 2018; Zieske and Schwille, 2014) suggesting a sensitivity of Min dynamics to protein concentrations. Visualization of the patterns was achieved by using fluorescently labeled proteins and flow chambers that establish gradients in Min protein concentrations. This made it possible to directly link the different protein concentrations to different arising patterns under otherwise identical experimental conditions. At concentrations most comparable to endogenous protein concentrations bursts were the dominating pattern (Vecchiarelli et al., 2016).

A main technical challenge of these reconstitutions is the use of supported lipid bilayers as a flat membrane surface. The supported lipid bilayers have a lower membrane-to-cytoplasm ratio than bacterial cells. As a result, the reconstitution on lipid bilayers requires high protein concentrations to study the Min-system dynamics. Vesicle- and microchamber-based reconstitutions, which more closely resemble the spatial constraints of bacterial cells and require much lower protein concentrations, more reliably recreate *in vivo*-like burst patterns (Caspi and Dekker, 2016; Zieske and Schwille, 2014). Additionally, the dynamics of the Min-system gradient can be affected by morphological changes of the membrane, especially around cytokinesis and septal closure, which are difficult to recreate *in vitro* (Di Ventura and Sourjik, 2011). *In vivo*, MinD and MinE dimers have reduced diffusion constants compared to *in vitro* studies, which could be due to molecular crowding within *E. coli* cells (Loose et al., 2008; Meacci et al., 2006). Addition of a crowding reagent (bovine serum albumin) facilitated the emergence of standing wave patterns of Min components inside microdroplets under conditions where no oscillations were observed in the absence of the crowder. This was hypothesized to be due to the crowder attenuating spontaneous binding of MinE to the membrane (Kohyama et al., 2019). Interestingly, artificially increasing MinD’s membrane affinity allowed for standing wave patterns even in a flat membrane system (Kretschmer et al., 2021). Combined, the Min patterns sensitivity to protein concentrations and membrane geometries have been major challenges for the *in vitro* reconstitution of the MinD/MinE oscillation to closely resemble *in vivo* dynamics.

**FIGURE 4:**
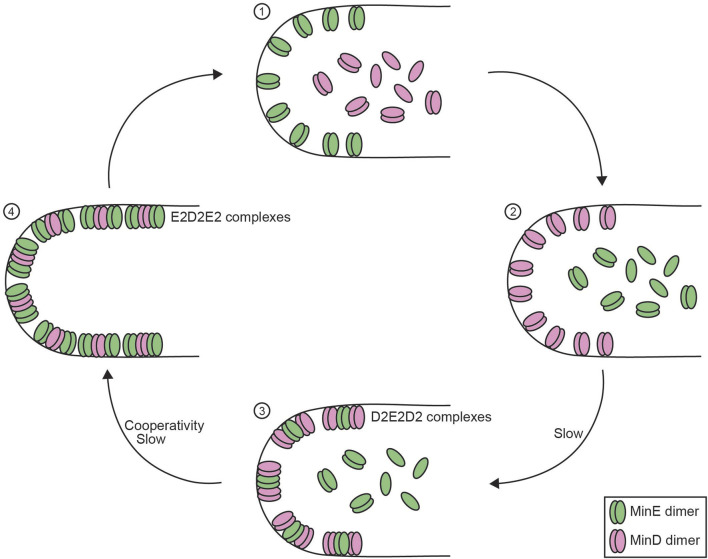
Reconstitution of the membrane-bound Min system. Schematic overview of the four-step process of the membrane-bound Min system in *E. coli* consisting of MinD and MinE proteins. (1) MinD monomers and dimers are diffusible in the cytoplasm whereas MinE dimers remain attached to the membrane. (2) MinE dimers release from the membrane, which allows MinD dimer to bind to the membrane. (3) MinE dimers interact with MinD, which allows MinE dimers to slowly bind to the membrane to form D_2_E_2_D_2_ complexes. (4) MinE dimers accumulate on the membrane and shift the balance to form E_2_D_2_E_2_ complexes, which causes the release of MinD dimers (Hu and Lutkenhaus, 2003). Negative feedback in this system is obtained as MinD facilitates the membrane integration of MinE, which stimulates MinD ATPase activity that results in MinD release from the membrane, thus MinD actively stimulating its own release. MinE dimers require a MinD-induced conformational change to bind the membrane (Park et al., 2011), which contributes to the time delay between MinD and MinE binding. Enrichment in MinE on the membrane that is required for MinD release generates cooperativity within the Min system (Mizuuchi and Vecchiarelli, 2018; Vecchiarelli et al., 2016).

Since its discovery, significant mechanistic details into this pole-to-pole oscillator have been uncovered (Figure 4). The MinD ATPase dimerizes in its ATP-bound state, which triggers a conformational change that alters the C-terminal helix to form a membrane targeting sequence (MTS) and anchor MinD to the membrane (Hu and Lutkenhaus, 2003; Wu et al., 2011). MinE, also a dimer, requires binding to MinD for a conformational change to expose its own MTS, bind the membrane, and form a complex of MinE and MinD dimers (D2E2 complex) (Park et al., 2011). Subsequently, more membrane-bound MinE, which interacts with D2E2 complexes forming E2D2E2 complexes, now stimulates the ATP hydrolysis activity of MinD, which causes MinD release from the membrane (Hu et al., 2002; Hu and Lutkenhaus, 2003). After MinD release, active MinE lingers for several seconds on the membrane before returning to the inactive form. During this time, the lingering MinE can bind with remaining D2E2 complexes on the membrane, which promotes the release of more MinD and form more lingering MinE thereby forming a positive feedback loop. At high density, lingering MinE can prevent MinD from rebinding regions that other MinD dimers have just dissociated from (Mizuuchi and Vecchiarelli, 2018). This defines the negative feedback between MinD and MinE. Additionally, the balance between the lingering MinE density and the concentration of active MinD dimers can act as a toggle switch, which establishes critical nonlinearity in the system (Vecchiarelli et al., 2016). Structural auto-inhibition of MinE ensures temporal segregation of MinD and MinE binding to the membrane and provides sufficient time delay within the oscillator. The relatively low MinD ATPase activity (∼1.5*10^3^ per day) triggering MinE release contributes to the time delay, and the rate of ATP hydrolysis has even been hypothesized to determine the time scale of Min dynamics (Ayed et al., 2017). Together, the interplay between MinD and MinE and temporal segregation of membrane binding creates oscillations on the membrane to generate a pole-to-pole gradient.

The Min-system example shows the success of *in vitro* reconstitution of a membrane-bound oscillator, however also implies unique challenges. The spatial dynamics of the Min system showed various patterns depending on the specific *in vitro* system. Switching from flat membrane systems to confined membrane systems with similar shape and volume to bacterial cells improved the similarity between *in vitro* and *in vivo* oscillation patterns. Adjusting protein concentrations to approximate *in vivo* levels allowed to establish a robust *in vitro* reconstitution with burst-like patterns of Min oscillations (Caspi and Dekker, 2016).

One main challenge ahead will be to link the Min system oscillation to its downstream targets to establish the cell division plane. So far, MinD/MinE oscillation reconstitutions have been unable to connect Min-system dynamics to the assembly of the Z-ring. Z-ring formation in *E. coli* is driven by FtsZ polymerization and the ring functions as an assembly platform for division proteins (Bi and Lutkenhaus, 1991). Previous efforts aimed to include Z-ring formation with MinD/MinE oscillation patterns (Di Ventura and Sourjik, 2011). To circumvent the need for FtsZ adaptors to link FtsZ to the membrane, FtsZ monomers were fused with MinD MTSs (Di Ventura and Sourjik, 2011). However, the FtsZ adaptors are the actual targets of MinC-mediated inhibition of Z-ring assembly (Hu et al., 1999; Wu and Errington, 2012). Without the FtsZ adaptors, the system could not establish the inhibitory link between MinC and FtsZ polymerization and this remains the major challenge for future reconstitutions to connect the Min system dynamics to Z-ring formation.

#### 2.4.3 Reconstitutions of transcription-translation oscillators: goodwin and beyond

Transcription-translation oscillators are conceptually intuitive, as mRNA and protein synthesis can easily introduce negative feedback and substantial time delays. However, their experimental reconstitution has proven challenging, primarily due to the difficulties in implementing transcription, translation, and degradation processes *in vitro*, as discussed earlier. In this section, we will highlight examples of synthetic transcription-translation oscillators that, despite these challenges, have successfully progressed from theoretical model to experimental reconstitution–and explore the insights gained from these achievements.

The Goodwin oscillator has been difficult to reconstitute partly due to the molecular challenge to implement the high Hill coefficient (>8) required for sustained oscillations. The closest *in vitro* reconstituted system is the two-switch negative feedback oscillator (Kim and Winfree, 2011). This oscillator consists of two synthetic DNA molecules with a regulatory segment, a promoter, and an output segment. One DNA molecule produces an RNA molecule that can be processed and eventually inhibits the expression of the second DNA molecule, where the second DNA molecule produces an RNA molecule that eventually activates expression of the first DNA molecule (Kim and Winfree, 2011). RNA degradation is facilitated by RNAse H. The system recreates the transcriptional negative feedback of the Goodwin oscillator, albeit diverging from the original design of the Goodwin oscillator by using two gene components instead of one. The design generated oscillations, but these dampened after only three cycles (Kim and Winfree, 2011). The authors hypothesized that the RNA degradation by RNAse H resulted in the build-up of short RNA fragments that interfered with the switch-to-switch feedback. Additionally, this system suffered from buffer exhaustion, e.g., magnesium ions, and resource depletion, e.g., NTPs, that could further explain the dampened limit cycle oscillations (Kim and Winfree, 2011). Based on their initial design, the authors also designed an *in vitro* repressilator system with reverse transcriptase, RNA polymerase, and DNA templates, which yielded oscillatory behavior in their simulations (Kim and Winfree, 2011). The *in vitro* reconstitution indeed displayed oscillations, though again, these dampened within three cycles, possibly due to DNA degradation and accumulation of proteins. Overall, this study showed the promise of *in vitro* reconstitution of genetic oscillators yet displayed problems with substrate depletion, waste accumulation, and adverse part degradation, which hindered sustained oscillations.

One approach to address substrate depletion and toxic byproduct accumulation is to shift from a batch system to flow systems. In flow systems, substrates can be replenished and byproducts removed either by diffusion–unrestrained (Karzbrun et al., 2014) or through a semi-permeable membrane (Hahn et al., 2007) – or by periodically diluting the reaction volume with fresh reaction mix, similar to a chemostat (Hansen et al., 2004; Niederholtmeyer et al., 2013). Both diffusion and dilution can also alleviate the need for active mRNA and protein degradation by continuously or periodically removing these macromolecules in a degradation-independent manner. An additional advantage of such microfluidic flow systems is that the dilution rate can be easily tuned allowing one to find conditions where synthesis and degradation rates are balanced, whereas fine-tuning the endogenous synthesis and degradation rates is much more challenging. In contrast to batch reactions, flow systems–operating far away from equilibrium–may even better reflect living cells by mimicking processes such as metabolite exchange across membranes and protein dilution during cellular growth.

Using a nanoliter-scale microfluidic reactor with discontinuous dilution, an extended version of the Goodwin oscillator–incorporating positive feedback–was successfully modeled and constructed *in vitro* based on the PURE system (Niederholtmeyer et al., 2013). The genetic oscillator comprised three DNA templates: T3 RNA polymerase (T3RNAP), the transcriptional repressor TetR, and *supD* amber suppressor tRNA, which prevents termination at UAG stop codons. T3RNAP transcribes the genes for the amber suppressor tRNA and *tetR* mRNA, and catalyzes its own expression, a form of positive feedback. Translation of *tetR* mRNA only occurs when enough amber suppressor tRNA is present, and once translated, TetR represses T3RNAP transcription, closing the negative feedback loop. Time delay originates from transcription and translation, as well as the time required for buildup of the amber suppressor tRNA. Cooperative binding of T3RNAP, *supD* amber suppressor tRNA, and TetR to their target sites is the source of nonlinearity. For sustained oscillations to occur, the model predicted that the dilution rate of this system should lie within a limited range (60 min > μ^−1^ > 100 min) with the range increasing with decreasing *supD* concentrations. This behavior was confirmed *in vitro*, although the actual dilution rates supporting oscillations were slightly lower than predicted. The oscillation period generally increased with the dilution rate, and stable oscillations were observed for up to 30 h (Niederholtmeyer et al., 2013). Together, these findings demonstrate that miniaturized discontinuous flow chambers can sustain genetic oscillations *in vitro* over extended periods of time by overcoming the product accumulation issues commonly encountered in batch reactions.

The development of an improved *E. coli*-based transcription–translation (TX-TL) system, along with versatile genetic toolboxes, accelerated the reconstitution of synthetic oscillators (Shin and Noireaux, 2012). A simple negative feedback oscillator–based on sigma factor σ28 activating the expression of the *cI* repressor and GFP, and *cI* in turn repressing the expression of σ28 – was engineered using a flow system with immobilized DNA in small reservoirs. Reactants were supplied via diffusion along a gradient through narrow channels, while products synthesized in the reservoir, such as GFP, diffused in the opposite direction (Karzbrun et al., 2014). The system produced oscillations with a period of 2.5 h, though the oscillations visibly dampened by the end of the experiment (12 h). Shortly thereafter, the classic repressilator was reconstituted *in vitro* using its original network design (Figure 2), combining discontinuous flow nanoreactors with the improved TX-TL system (Niederholtmeyer et al., 2015). This setup generated sustained oscillations for at least 50 h, with a period matching those observed *in vivo*–a major improvement over previous efforts, where damped oscillations were observed after just 10 h (Kim and Winfree, 2011; Karzbrun et al., 2014).

Negative feedback is implemented in the repressilator model through the odd number of repressive interactions. Theory suggests that in the absence of noise only repressilator systems with an odd number of nodes oscillate, whereas those with an even number of nodes are mono- or multistable (Fraser and Tiwari, 1974; Smith, 1987). Indeed, a constructed four-nodes circuit did not exhibit oscillations whereas a novel five-nodes synthetic repressilator design did (Niederholtmeyer et al., 2015). Furthermore, chemical perturbations of the four-nodes circuit allowed the system to switch between two stable steady-states. These experiments not only align with the theoretical predictions, but also confirm that the observed oscillations originate from the network’s design rather than artifacts of the microfluidic setup. In the repressilator model, nonlinearity is thought to arise from the dimeric nature of the transcriptional repressors as well as the presence of two repressor binding sites in each promoter (Elowitz and Leibler, 2000). Removing one of the two repressor binding sites in the reconstituted repressilator resulted in either dampened oscillation, or complete loss of oscillatory dynamics, highlighting the necessity for sufficient nonlinearity (Niederholtmeyer et al., 2015). Balanced synthesis and degradation rates were similarly important: Lower amounts of DNA template–a proxy of lower mRNA and protein synthesis rates–required lower dilution rates in order to show oscillations. Similarly, increased protein degradation by fusing strong ssrA-tags to the transcriptional repressors increased the oscillation period and required lower dilution rates to allow for oscillations.

Together, these studies highlight the potential of *in vitro* reconstitution for in-depth investigation of oscillator dynamics and its underlying molecular determinants, but also reveal its challenges. The *in vitro* platforms furthermore facilitate efficient prototyping of novel circuit designs and have subsequently been used for the optimization of the two-component negative feedback oscillator to yield more robust, sustained oscillations (Yelleswarapu et al., 2018; Tayar et al., 2017).

## 3 Conclusion

Here, we have discussed the theoretical requirements, experimental challenges and eventual successes of reconstituting various biological oscillators. The reconstitution provided novel insights into the molecular interactions and dynamics of the systems, as well as valuable lessons in how to overcome experimental challenges–some unique to oscillators, others broadly relevant to reconstitution studies in general.

The cyanobacterial circadian oscillator was the first biological oscillator successfully reconstituted *in vitro*. As a post-translational oscillator, it operates independently of transcription and translation, thereby avoiding several common reconstitution challenges. Remarkably minimalistic, the system only requires purification of three core proteins–KaiA, KaiB, and KaiC–with the phosphorylation and dephosphorylation cycle driven by a single complex, the KaiC hexamer. Despite its simplicity, identifying the minimal configuration, precise protein concentrations, stoichiometries, and experimental conditions necessary for sustained oscillations posed a significant challenge–particularly given the oscillator’s long period, which required sampling every 2 hours over 3 days. The initial reconstitution using just the three core proteins produced rhythms with reduced amplitude and a shorter period compared to *in vivo* observations (Nakajima et al., 2005). Subsequent high-throughput approaches with automated data acquisition enabled precise determination of the narrow parameter ranges and molecular interactions required for full circadian clock function. These advances, along with the inclusion of additional downstream regulators, allowed for a more accurate and complete reconstitution of the oscillator (Nakajima et al., 2010; Kageyama et al., 2006; Gutu and O'Shea, 2013; Kaur et al., 2019; Chavan et al., 2021). Even system level behaviors including temperature compensation and entrainment could be reproduced (Nakajima et al., 2005; Rust et al., 2007), and ancient circadian clocks reconstructed.

In our example of a membrane-bound oscillator–the Min system–both compartment- and membrane-specific challenges arose along with a striking sensitivity to protein concentrations. *In vitro* reconstitution of Min-system dynamics revealed a wide variety of spatial patterns, in contrast to the burst-like standing waves typically observed *in vivo*. Using a flow cell with a supported lipid bilayer to create protein gradients, researchers systematically mapped the dependence of pattern formation on protein concentration and confirmed that bursts dominate at endogenous protein levels (Vecchiarelli et al., 2016), when MinD concentration becomes limiting. Interestingly, membrane composition might also influence oscillation behavior: Cooperative binding of MinD to liposomal membranes, with Hill coefficients ranging from 1.5 to 2.5 depending on membrane composition, has been reported (Mileykovskaya et al., 2003; Renner and Weibel, 2012). Similarly, geometry and reaction volume are of critical importance in this system. Only improved reconstitution systems working with confined microchambers using geometries and volumes reminiscent of bacterial cells were able to consistently generate bursts and standing waves similar to the observed *in vivo* dynamics (Caspi and Dekker, 2016; Zieske and Schwille, 2014). These *in vitro* reconstitutions mark a major step toward reconstructing the entire cell division machinery, potentially facilitating symmetric division of a synthetic cell.

From the reconstitution efforts of transcription-translation oscillators, several key challenges emerged: The requirement for fast, high-yield cell-free protein synthesis, the need for balanced protein degradation or dilution, and the poisoning of the system through the accumulation of toxic waste products. Furthermore, initial *in vitro* reconstitutions suffered from nonspecific nuclease activity. Improved CFPS systems based on *E. coli* lysate or the PURE system combined with microfluidic flow systems allowed for the successful reconstitution of several synthetic oscillators. The flow chambers enabled continuous exchange of reagents–supplying fresh substrates while efficiently removing toxic byproducts and diluting proteins and mRNA (Niederholtmeyer et al., 2013; Karzbrun et al., 2014). In that way, the problem of product accumulation was overcome without the need to reconstitute the proteolytic degradation machinery. The optimized systems have been successfully used to efficiently prototype novel circuits and oscillator networks (Niederholtmeyer et al., 2015; Yelleswarapu et al., 2018; Tayar et al., 2017). Overall, these examples underscore the power of *in vitro* reconstitution for uncovering the molecular interactions and biophysical principles underlying biological oscillators, while also highlighting its potential for bioengineering and synthetic biology applications.

### 3.1 Future perspective

*In vitro* reconstitution offers a powerful approach to precisely define the essential components and parameters governing oscillatory dynamics. However, effectively navigating the multidimensional parameter space continues to present a significant challenge. As exemplified by the Kai and Min systems, suboptimal protein concentrations can dampen or preclude the generation of sustained oscillations, underscoring the importance of parameter optimization. In both systems, the ability to efficiently screen concentration ranges, stoichiometries and experimental conditions significantly contributed to the successful parameter identification and a deeper understanding of the underlying molecular mechanisms. Consequently, future efforts in *in vitro* reconstitution of biological oscillators will significantly benefit from the incorporation of high-throughput screening methodologies to more effectively explore the relevant parameter spaces. These include ultrahigh throughput droplet microfluidics (Gan et al., 2022; Hori et al., 2017) and miniature chemostats (Swank and Maerkl, 2021), and will require compatible–likely fluorescent–readouts such as fluorescent probes, dye-labeled proteins, or appropriate biosensors, that can be monitored and analyzed (semi-)automatically.

Another key challenge that remains in the *in vitro* reconstitution of biological oscillators is satisfying their energy demands for sustained oscillations. All successful reconstitutions discussed here rely on external energy sources, though the extent of this dependency varies depending on the type of oscillator and network architecture. In the most recent reconstitution of the Kai system, ATP consumption by the KaiC ATPase was estimated at 20–26 ATP molecules per KaiC monomer per day (Chavan et al., 2021), though this figure excludes ATP usage by the output kinases. Considering that all proteins were present at concentrations between 1–4 μM, the supplied 1 mM ATP should be able to promote oscillations in a batch system for at least a week.

For the most recent reconstitutions of the Min system, the energy consumption was not precisely measured, however, the MinD dimer primarily binds the membrane in the ATP-bound state and releases after ATP hydrolysis. From this knowledge, one can extrapolate that each time a MinD dimer binds to and releases from the membrane it requires two molecules of ATP. For the *in vitro* Min system, approximately 1 µM of MinD and MinE were injected with 5 µM of ATP in the presence of a phospho (enol)pyruvic acid (PEP, 5 µM) and pyruvate kinase as a ATP-regeneration system (Caspi and Dekker, 2016). Although this setup presents a smaller molar excess of ATP compared to the Kai system, the ATP regeneration system compensates for this difference.

However, these energy demands are dwarfed by the energy requirements of genetic oscillators driven by the energetic burden of transcription, translation, and protein degradation. Polymerase-dependent transcription requires little energy, however, the demands for the synthesis and recycling of NTPs is significant: NTP synthesis requires ∼30 ATP per nucleotide (Hu et al., 2020), whereas the cost of recharging and recycling NTPs sum to two ATP per base (Lynch and Marinov, 2015). Current CFPS systems circumvent these energy costs by providing the NTPs in the reaction mix. Protein synthesis requires ∼4-5 ATP per amino acid, considering loading of the amino acids onto tRNA and the ribosomal elongation process (Hu et al., 2020; Lynch and Marinov, 2015; Princiotta et al., 2003). Similarly, ClpXP-mediated protein degradation was determined to require between 0.3-4 ATP per amino acid, depending on the stability of the protein (Kenniston et al., 2003). The *in vitro* repressilator cycled with a period of about 2–3 h, where each of the three protein peaks ranged between 0.1–2 µM with a combined total peak value of ∼3 µM (Niederholtmeyer et al., 2015). Considering the amount of protein made, and the energy requirement for synthesis and degradation, the energy required for repressilator oscillations is orders of magnitudes higher than the consumption of the Kai and Min systems. To overcome the high energy burden of protein synthesis and degradation, all successful genetic oscillator reconstitutions so far have used flow chambers providing continuous energy, in the form of NTPs, removing waste products and diluting out mRNA and proteins to reduce the energetic burden imposed by protein degradation. Clearly, the protein degradation problem has not been solved, though at least in one study expression of additional ClpXP in an *E. coli* lysate was used to improve oscillation capacity (Tayar et al., 2017). Incorporating CFPS reactions into liposomes, giant unilamellar vesicles (GUVs), or hydrogel-based beads might enable the confinement of proteins, mRNA, and DNA, while still allowing the exchange of small molecules such as ATP and amino acids (Van de Cauter et al., 2021; Zhou et al., 2018). Such setups could potentially support a continuous supply of energy and substrates from an outside reservoir, helping to meet the demands of energy-intensive oscillators, while also keeping the volumes of the reactions to biologically relevant scales.

An alternative to the continuous external supply of ATP is the incorporation of an ATP regeneration system directly into the *in vitro* system. ATP regeneration systems enzymatically recycle adenosine di- or mono-phosphate (ADP or AMP) arising during the biochemical reaction back to ATP using high-energy phosphate donors such as PEP or creatine phosphate. In contrast to the simple addition of ATP at the start or in a discontinuous manner, such systems keep the ATP concentration constant and ADP–inhibitory to many enzymatic reactions–low over extended periods of time. For example, Min-system reconstitutions used PEP and pyruvate kinase to regenerate ATP (Caspi and Dekker, 2016). Similarly, the PURE system contains creatine and creatine kinase to regenerate ATP (Shimizu et al., 2001). However, while these systems maintain stable ATP and ADP concentrations, they still result in the accumulation of inorganic phosphate, which can precipitate magnesium ions–an essential cofactor for many enzymatic reactions, and therefore poison the system. Consequently, increasing the recycling of inorganic phosphate through maltose addition, significantly increased the protein yields of the *E. coli-*based TX-TL system (Caschera and Noireaux, 2014). Recently, the integration of a pathway that recycles inorganic phosphate under consumption of pyruvate and oxygen to regenerate ATP was shown to enhance the protein yield of the PURE system by up to 78% (Yadav et al., 2025). Another alternative could be light-driven ATP synthesis (Steinberg-Yfrach et al., 1998; Lee et al., 2018; Berhanu et al., 2019). This approach incorporates a small photosynthetic organelle, containing in its membrane a light-driven proton pump (such as proteorhodopsin or bacteriorhodopsin) and a proton-gradient driven ATP synthase, into giant unilamellar vesicles to generate ATP under light exposure. Such a system has been shown to produce sufficient ATP to allow for the *de novo* protein synthesis of its energy-producing components (Berhanu et al., 2019). These promising examples highlight the prospect of more efficient energy-producing systems to satisfy the energy demand of transcription- and translation-dependent oscillators.

Improved and efficient reconstitution methodologies will enable further in-depth investigations into the intricate properties of these dynamic systems–including tunability, robustness, entrainability, temperature compensation, coupling, and their adaptation to cellular growth; properties that have been extensively studied theoretically, but their effects have been difficult to isolate in cellular or *in vivo* systems.

A particularly interesting aspect is the robustness of biological oscillators against molecular noise. Many naturally occurring biological oscillators are thought to be highly robust. Addition of positive and negative feedback have been proposed as mechanisms to protect oscillatory systems against molecular noise, thereby supporting more robust oscillations across a broader range of parameters (Tsai et al., 2008; Qiao et al., 2022). On the other hand, it has also been shown that stochastic noise can facilitate oscillations, so called noise-induced or noise-seeded oscillations, beyond the limits of the deterministically possible–albeit they are usually more irregular in amplitude and frequency (Fraser and Tiwari, 1974; Vilar et al., 2002; McKane and Newman, 2005; Purcell et al., 2010). With cellular processes being inherently noisy and stochastic, rather than deterministic, there is an increasing interest in understanding the impact of biological noise on oscillations as well as mechanisms that buffer against biological noise in a systematic fashion using reconstituted systems.

However, most reconstituted oscillators studied to date have been examined in rather large volumes (in the microliter range) and in systems with limited capacity for performing the number of replicates necessary to study such stochastic processes. Reconstitution of oscillators in small droplets, membrane-encapsulated vesicles, or miniaturized (flow) chambers with femtoliter- to picoliter-scale volumes can facilitate the parallel measurement of hundreds or thousands of individual oscillatory reactions at volumes and copy numbers relevant to cellular systems. Introducing stochastic or engineered variability in the distribution of components across droplets can enable efficient exploration of the parameter space, quantification of variability, and identification of the sources and effects of biological noise. Such an approach has been successfully taken to study dependencies, robustness, and temperature compensation of the embryonic cell cycle oscillator by encapsulating cycling frog egg extracts in water-in-oil droplets (Rombouts et al., 2025).

One example, where such an approach has been applied, is the study from Tayar et al.: They reconstituted a two-component negative feedback oscillator with or without additional stabilizing negative feedback in 50 individual microcompartments (Tayar et al., 2017), and showed that period variability was decreased in the presence of the stabilizing feedback. Similar studies examining different network architectures *in vitro* and how they amplify or suppress noise would extend and refine extensive *in silico* studies on this topic (Qiao et al., 2022; Tsai et al., 2008; Gonze et al., 2002). For example, it would be interesting to explore the precise contribution of positive feedback implemented in a system such as the genetic negative plus positive feedback oscillator presented by Niederholtmeyer et al., in 2013.

Another prime example is the cyanobacterial circadian clock, which is thought to have near perfect precision in *S. elongatus* (Eremina et al., 2025). However, *in vivo* and *in silico* analyses suggest that the circadian clock is surprisingly sensitive to copy number changes, as fluctuations in molecule abundances propagate and amplify due to the delayed negative feedback loop (Chew et al., 2018). This is consistent with the narrow stoichiometric boundaries found in *in vitro* reconstitutions, which allowed for sustained oscillations. In a recent study, the KaiABC oscillator was encapsulated into femtoliter-sized giant unilamellar vesicles (GUVs, (Li A. Z. T. et al., 2025)). This enabled the reconstitution of the cyanobacterial circadian oscillator in thousands of vesicles, with volumes, concentration ranges, and variability comparable to those found in cells. Strikingly, the concentrations commonly used in bulk *in vitro* reconstitutions of the KaiABC oscillator produced robust oscillations only in larger volumes, not in GUVs with volumes similar to *S. elongatus*. Increased protein concentrations rescued the oscillations. The required higher concentrations of Kai proteins are consistent with the determined *in vivo* concentrations and suggest that the high protein concentrations are important to ensure high-fidelity oscillations (Chew et al., 2018; Li A. Z. T. et al., 2025) potentially due to KaiB being partially localized to the GUV membrane, and therefore removed from the reaction pool. These reconstitutions further emphasized that the KaiABC system is highly robust at endogenous protein concentrations in the absence of any additional feedback, highlighting the power of *in vitro* reconstitutions to dissect such dependencies. Interestingly, the multicellular filamentous cyanobacterium *Anabaena* sp. PCC 7120 may maintain robust circadian oscillations despite low copy numbers and high molecular noise by coupling cells through septal junctions. In theory, such coupling of noisy oscillators can sustain stable oscillations even outside the deterministic parameter range, relying critically on the system’s inherent stochasticity (Arbel-Goren et al., 2021).

*In vivo*, biological oscillators must be robust despite constraints imposed by the cellular environment, such as growth and division. In a growing cell, simply maintaining constant concentrations of oscillator components requires their continuous synthesis. However, all newly synthesized components enter the oscillator circuit in their nascent, unmodified state and might therefore change the balance of components and oscillatory dynamics. Conversely, without ongoing synthesis, protein concentrations decrease in a growing cell due to dilution–even in the absence of targeted degradation–which can also affect oscillatory behavior. Another interesting case is DNA replication, which introduces discrete two-fold changes in the number of activator or repressor binding sites. Such stepwise perturbations must likewise be accommodated in transcription-translation oscillators to preserve stable oscillations *in vivo*. Spike-in or dilution experiments, conducted either in batch reactions or using microfluidic flow chambers, could offer valuable insights into how oscillators respond and adapt to these fluctuations. Clearly, there are still many open questions to be answered.

So far, the oscillators discussed here are derived from unicellular systems, or, in the case of the transcription-translation oscillators, from synthetic systems. However, many multicellular systems exhibit oscillations across membranes, cell populations and tissues, which introduces additional layers of complexity in systems behaviour including coupling and synchronization, and spatio-temporal phenomena such as waves (Jimenez et al., 2022). Developing experimental setups where such complex, multi-oscillator behaviours can be studied and dissected *in vitro* would be an exciting advance. Tayar et al. have taken a first step in this direction by connecting their array of microcompartments through channels, which allows diffusion and coupling between oscillators. Indeed, this method successfully recapitulated oscillator synchronization and complex behaviors such as pattern formation and symmetry breaking (Tayar et al., 2017). Similarly, within the cellular context, biological oscillators commonly interact and synchronize with other external or internal oscillators through entrainment or gating (Jimenez et al., 2022). For example, the cyanobacterial circadian clock is entrained by light, where the light-dark oscillation caused by the Earth’s rotation is coupled to the KaiC phosphorylation state. Entrainment of *S. elongatus* is thought to be mediated by changes in the cellular ATP/ADP ratio (Rust et al., 2011) and the redox state–specifically, the levels of oxidized quinone (Kim et al., 2012) – in response to photosynthetic activity during the day. Recently, the contribution of individual components to the entrainment of the cyanobacterial circadian clock has been elegantly dissected using the *in vitro* reconstituted system. Using high-troughput measurements, the phase-depent response of the *in vitro* clock to perturbations (addition of quinone or ADP) was assessed in the presence or absence of different clock components, revealing an intricate interplay between the core KaiABC oscillator and the output kinases CikA and SasA (Fang et al., 2023), a conclusion which would have been difficult to draw from the cellular system. These examples underline the potential of *in vitro* reconstitution to reveal fundamental principles of collective dynamics.

In summary, *in vitro* reconstitution of biological oscillators has become a powerful approach for uncovering the molecular mechanisms, dynamic properties, and hidden requirements of these fascinating molecular clocks. Recent advances in synthetic biology–particularly the miniaturization of flow systems, automation of experimental workflows, and improvements in CFPS systems–have addressed key technical challenges and significantly accelerated progress in the field. At the same time, the standardized toolkits and engineering mindset of synthetic biology have shifted much of the focus toward designing and optimizing novel synthetic networks, rather than probing the inner workings of naturally occurring oscillators. These efforts also converge with another overarching goal of synthetic biology, building a synthetic cell–one that can autonomously regulate and coordinate the temporal phases of growth and division potentially by incorporating a biological oscillator. While this direction is undoubtedly promising, it will be equally exciting to now redirect these emerging technologies and insights back toward fundamental cellular oscillators–such as the cell cycle or the somatic segmentation clock–to achieve a level of mechanistic understanding comparable to what has been accomplished for the cyanobacterial circadian clock. Either way, the era of *in vitro* reconstitutions of biological oscillators might have just begun.
